# The Wear Behavior of Glass-Ceramic CAD/CAM Blocks against Bovine Enamel

**DOI:** 10.3390/ma16216839

**Published:** 2023-10-24

**Authors:** Tomoko Someya, Masaaki Kasahara, Shinji Takemoto, Masayuki Hattori

**Affiliations:** 1 Department of Dental Materials Science, Tokyo Dental College, 2-9-18 Kandamisaki-cho, Chiyoda-ku, Tokyo 101-0061, Japan; kasaharamasaaki@tdc.ac.jp (M.K.); hattori@tdc.ac.jp (M.H.); 2 Department of Biomedical Engineering, Iwate Medical University, 1-1-1 Idaidori, Yahaba-cho, Iwate, Shiwa-gun 028-3694, Japan; takemoto@iwate-med.ac.jp

**Keywords:** two-body wear test, glass-ceramic, bovine enamel, wear behavior, CAD/CAM blocks

## Abstract

The wear of enamel and crown restorative materials often occur by occlusion. The purpose of this study was to evaluate the wear volume between glass-ceramics used for CAD/CAM blocks (lithium disilicate: Initial LiSi block (LIS), IPS e.max CAD (IPS), zirconia-reinforced lithium silicate glass-ceramics: Celtra DUO (DUO), VITA Suprinity (VITS) and feldspar-based glass-ceramics: Vitablocs Mark II (MAK)) and bovine tooth enamel using a two-body wear test, the hardness, three-point bending strength, micro-structure and the element components of glass-ceramics. The data were analyzed using a one-way analysis of variance and Tukey’s multiple comparison test (α = 0.05). IPS and DUO with relatively large size crystal gain had significantly larger abrader wear volumes. Zirconia-reinforced lithium silicate glass-ceramics (DUO, VITS) caused significantly greater wear volume in antagonist enamel. MAK with scale-shape crystals grains produced distinct scratches after wear tests, both in the material itself and in the enamel. A strong correlation between the mechanical properties (hardness, three-point bending strength) and wear volume could not be confirmed. The type of glass-ceramic, size, and shape of the crystal grains affected the wear behavior of the glass-ceramics for CAD/CAM blocks. Therefore, dentists should consider that wear behavior varies with crystal structure, size, and shape in glass-ceramics for CAD/CAM blocks.

## 1. Introduction

The advancement of digital dentistry through the use of computer-aided design/computer-aided manufacturing (CAD/CAM) technology has increased the selection of manufacturing methods for dental prostheses. CAM can be divided into additive and subtractive manufacturing methods [1,2,3,4]. When fabricating objects with complicated structures, less waste can be expected with the additive manufacturing method compared to the subtractive manufacturing method. However, post-processing work is troublesome for additive manufacturing because the support materials must be removed after processing [4,5,6]. For the subtractive manufacturing method, prosthetic devices are machined from a uniform material (CAD/CAM block or disc). This method enables the fabrication of prostheses with fewer defects than ones made via conventional manual manufacturing [7,8]. In addition, the fitting accuracy is higher than that of prosthetic devices manufactured using the additive manufacturing method, and especially for crowns, the subtractive manufacturing has become the prominent method used in clinical practice due to the increased use of milling devices for CAD/CAM blocks [8,9].

The materials for CAD/CAM blocks used for subtractive manufacturing to fabricate crowns include metals, ceramics, polymers, and composites [4,10]. Among them, ceramics are highly aesthetic and possess the ideal mechanical properties, chemical stability, and biocompatibility [11,12]. Feldspar-based glass-ceramics are made by adding feldspar to alumina silicate and have been used as block materials since the introduction of CAD/CAM technology to the dental field [13,14]. Lithium disilicate glass-ceramics are used the most for CAD/CAM blocks, and have a structure in which lithium disilicate crystals are randomly arranged in the glass layer (Li_2_O/2SiO_2_ glass phase) [13,14]. Zirconia-reinforced lithium silicate glass-ceramics are a mixture of lithium silicate with approximately 10% zirconia, and the material is considered to have better mechanical strength than lithium disilicate glass-ceramics [13,14]. Finally, zirconia is also available as a material for CAD/CAM blocks in semi-sintered form. Zirconia possesses higher mechanical strength than other CAD/CAM ceramics; however, aesthetic issues remain problematic due to its low transparency [14,15].

Requirements for crown restoration materials include cost-effectiveness, usability, fracture resistance, and aesthetics [16]. In the oral cavity, wear caused by occlusion is an issue; therefore, wear resistance is also an essential requirement for use as crown restoration materials. The wear of the crown restoration materials induces occlusal changes, poor aesthetics, and poor oral function, which impedes clinical success [16].

For the long-term use of crowns in the oral cavity, the wear of opposing teeth must be considered in addition to the wear of the material itself. There have been various reports on the wear behavior of opposing teeth for crown restoration materials. Wille et al. reported that polymer-infiltrated reinforced glass networks and lithium disilicate glass-ceramics cause more wear on the antagonist’s teeth than do zirconia-reinforced lithium silicate glass-ceramics [17]. When zirconia or lithium disilicate glass-ceramics are used as the crown restoration material, the wear of the antagonist enamel was more significant when compared to the wear induced by resin composite for crowns [18]. A factor that determines the wear characteristics of the opposing teeth is the hardness of the material. Reports have suggested that crown restoration materials with higher hardness promote the wear of opposing teeth, while other reports have suggested that crown restoration material hardness and wear are independent from one another [17,19,20]. For a resin composite, a heterogeneous material composed of a resin matrix and fillers with fine structures, the size and shape of the filler particles affect the wear of the opposing teeth and the material itself [21,22,23,24,25,26,27]. Since the glass-ceramics used in CAD/CAM blocks are also composed of crystal and amorphous elements, they are considered heterogeneous, similar to resin composites [14,17]. The data reveals the causality between composite and ceramic materials’ structure, composition, and mechanical properties, and material wear. However, this data is from a limited subset, comparing distinct materials such as glass-ceramics, zirconia, and resin composites [17,18,28,29]. When investigating the relationship between the characteristics of ceramics and wear behavior, it is necessary to make a comparative study between ceramics with similar components.

Therefore, this study focused on the wear behavior of glass-ceramics for CAD/CAM blocks. The wear behavior of the glass-ceramic CAD/CAM blocks to antagonist bovine tooth enamel was evaluated using a two-body wear test. And the hardness, three-point bending strength, microstructure, and elemental components of glass-ceramic CAD/CAM blocks was investigated to clarify factors affecting wear behavior of glass-ceramic CAD/CAM blocks. The null hypothesis was that the mechanical properties (bending strength, hardness) of the glass-ceramic CAD/CAM blocks do not affect the wear volume of bovine enamel or the material itself.

## 2. Materials and Methods

### 2.1. Materials and a Flowchart

**Figure 1:**
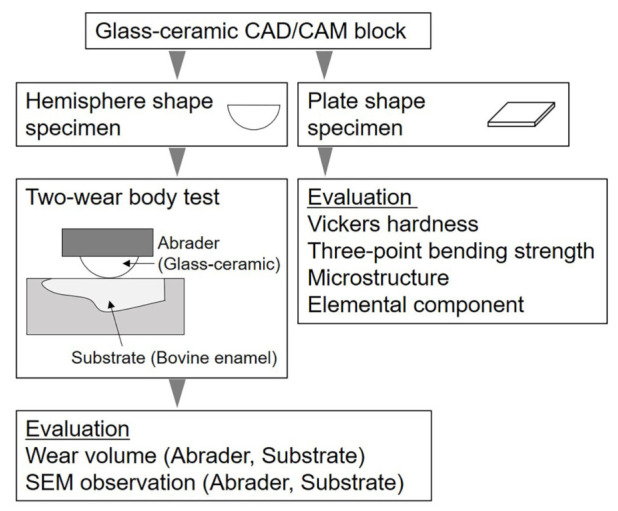
Flow chart of this study.

Table 1 shows the types of glass-ceramic CAD/CAM blocks used. In this study, two types of lithium disilicate glass-ceramics, two types of zirconia-reinforced lithium silicate glass-ceramics, and one type of feldspar-based glass-ceramic were used. Figure 1 shows a flow chart of this study.

### 2.2. Evaluation of Mechanical Properties of Glass-Ceramic CAD/CAM Blocks

#### 2.2.1. Vickers Hardness

With a low-speed cutting machine, glass-ceramic CAD/CAM blocks were cut to 2 mm thicknesses (ISOMET-LS, Buehler Japan, Tokyo, Japan). After cutting, IPS and VITS specimens were fired according to the instructions specified by the manufacturer. The specimens were polished to #1200-grid using water-resistant abrasive paper (*n* = 5). The hardness of each sample was measured using a Vickers hardness tester (MVF-G, Shimadzu, Kyoto, Japan) under a load of 4.9 N and a loading time of 20 s.

#### 2.2.2. Three-Point Bending Strength

Glass-ceramic CAD/CAM blocks were cut using a low-speed cutting machine and polished using water-resistant abrasive papers to #1200-grid so that each specimen had a thickness of 1.2 mm, a width of 4.0 mm, and a length of 14.0 mm (*n* = 6). IPS and VITS specimens were fired according to the instructions specified by the manufacturer. The specimens were placed on a three-point bending test jig, and a bending test was performed using a universal material testing machine (EZ-graph, Shimadzu, Kyoto, Japan). The distance between the support span was 10 mm, and the crosshead speed was 0.5 mm/min. The three-point bending strength was calculated from the maximum fracture load.

#### 2.2.3. Microstructure and Elemental Components of Glass-Ceramic CAD/CAM Blocks

A low-speed cutting machine was used to cut the glass-ceramic CAD/CAM blocks into 2 mm thicknesses. IPS and VITS were fired according to the manufacturer’s instructions. The specimens were polished to #1200-grid using water-resistant abrasive papers then placed in a plastic container containing 5 mol/L of sodium hydroxide solution, which was stored in a warm bath at 60 °C for seven days (*n* = 2). The specimens were then ultrasonically cleaned in distilled water and then coated with Au-Pd (Au: 60%, Pd: 40%). The microstructure was observed with a scanning electron microscope (SEM: SU-6600, HITACHI, Tokyo, Japan) on two spots per specimen.

Specimens were prepared using the same method as the SEM samples, and the crystalline structure was analyzed using an X-ray diffractometer (Ultima, Rigaku, Tokyo, Japan, Cu-Kα, 40 kV, 40 mA) with a semiconductor detector. After carbon-coating the specimens, the elemental components were analyzed on a typical spot for each material by an electron probe microanalyzer (*n* = 1, EPMA: JXA-8200, JEOL, Tokyo, Japan).

### 2.3. Wear Volume

#### 2.3.1. Preparation of Specimens

In this study, a two-body wear test was performed using glass-ceramic CAD/CAM blocks as the abrader specimen and bovine enamel as the substrate specimen, and the wear volume was measured. Each specimen of the glass-ceramic CAD/CAM block was machined into a hemisphere with a radius of 5 mm using a milling machine (CEREC MCXL, Dentsply Sirona K.K., Tokyo, Japan, software: CEREC SW v4.5.2, mode: fine). IPS and VITS were fired according to the manufacturer’s instructions. Each specimen was polished with dental abrasives (diamond silicon polisher blue/yellow, Dedeco, NY, USA) to use as abrader specimens. The arithmetic mean surface height (Sa) was measured as the surface roughness using a 3D laser microscope (Lext OLS 4000, Olympus, Tokyo, Japan, *n* = 3).

The substrate specimens were frozen-preserved bovine teeth thawed and cut at cement enamel junction using a low-speed cutting machine. After removing the pulp of the crown with dental tweezers, the tooth was embedded in an epoxy ring with a diameter of 1 inch with epoxy resin. After exposing the enamel with water-resistant abrasive paper, the surface was polished to # 1200-grid.

#### 2.3.2. Two-Body Wear Test

The abrasive and substrate specimens were attached to the wear tester, and a two-body wear test was performed in distilled water (*n* = 6). Measurements were taken after 30,000 strokes, where the stroke width was 3 mm, the speed was 1.5 Hz, and the load was 10 N.

**Figure 2:**
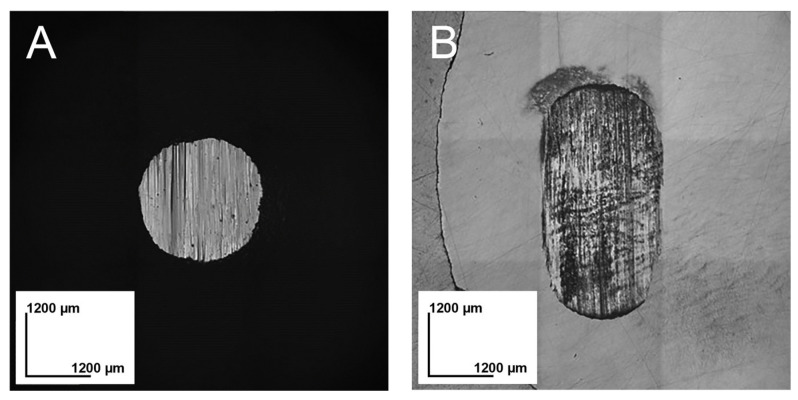
3D laser microscope images after the wear test: (**A**) abrader specimen (glass-ceramic CAD/CAM block), (**B**) substrate specimen (bovine enamel).

For the abrasive specimens, the diameters of the wore surface (Figure 2A) were measured at two points (vertical and horizontal) with a 3D laser microscope, and the average value was calculated. The wear volume was calculated using the following Formula (1).

V = 1/6πh(3c^2^ + h^2^) h = r − (r^2^ − c^2^)^1/2^ (1)

V: wear volume, r: radius of the hemispherical specimen (5 mm), c: radius of the worn circular surface, h: worn height of the hemispherical specimen 

For the substrate specimens, the worn surfaces (Figure 2B) were photographed using a 3D laser microscope, and the wear volume was calculated by integrating the data obtained from the images. In addition, the sample after the two-body wear test was observed by SEM after carbon coating.

### 2.4. Statistical Analysis

After a one-way analysis of variance (ANOVA) of Vickers hardness (*n* = 5), three-point bending strength (*n* = 6), average surface roughness (Sa, *n* = 3) of the abrasive specimens before the two-body wear test, and wear volume after the two-body wear test (abrader specimen, substrate specimen, *n* = 6), the data were analyzed using Tukey’s multiple comparison test (α = 0.05, BellCurve for Excel, Social Survey Research Information, Tokyo, Japan).

## 3. Results

### 3.1. Vickers Hardness

**Figure 3:**
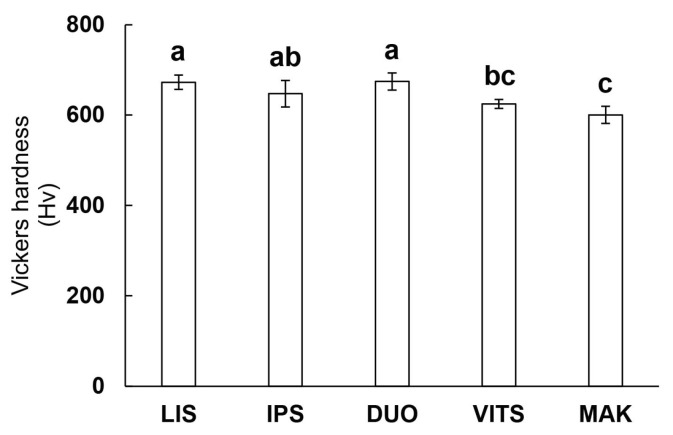
Vickers hardness. The same lowercase letters in the figure indicate no significant differences (*p* > 0.05, ANOVA and Tukey’s multiple comparison test).

Figure 3 shows the Vickers hardness (Hv) of each glass-ceramic CAD/CAM block. Vickers hardness was around 600 to 700 for all groups. According to the statistical analysis, the hardness of LIS and DUO was significantly higher than that of VITS and MAK (*p* < 0.05). The hardness of IPS was significantly higher than that of MAK. There were no differences between LIS, IPS, and DUO, between IPS and VITS, and between VITS and MAK (*p* > 0.05).

### 3.2. Three-Point Bending Strength

**Figure 4:**
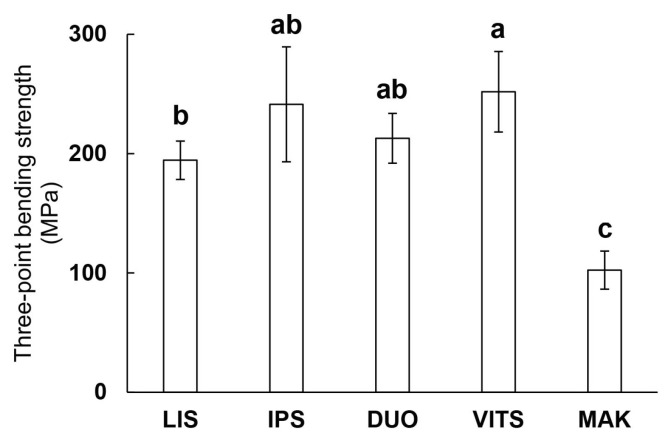
Three-point bending strength. Same lowercases in figure indicated no significant difference (*p* > 0.05, ANOVA and Tukey’s multiple comparison test).

Figure 4 shows the three-point bending strength of the glass-ceramic CAD/CAM blocks. The bending strength of MAK was about 100 MPa, which was significantly lower than that of the other groups (190 MPa or more) (*p* < 0.05). In addition, LIS had significantly lower bending strength than VITS (*p* < 0.05). There were no significant differences between IPS, DUO, and VITS, and between LIS, IPS, and DUO (*p* > 0.05).

### 3.3. Microstructure and Elemental Components

**Figure 5:**
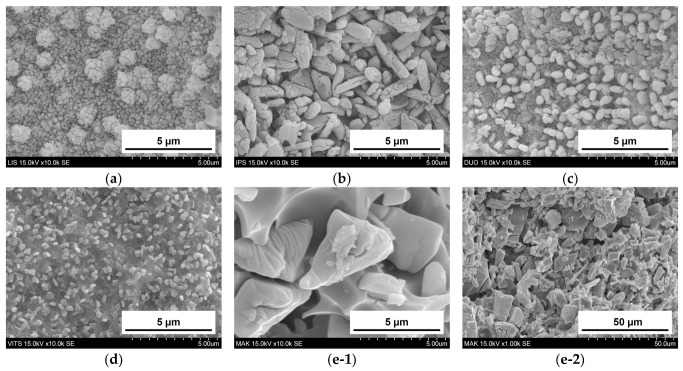
SEM images of glass-ceramic CAD/CAM blocks: (**a**) LIS (**b**) IPS (**c**) DUO (**d**) VITS (**e-1**) MAK (10,000× magnification), significantly enlarged (**e-2**) MAK slightly enlarged (1000× magnification).

Figure 5 shows SEM images of the glass-ceramic CAD/CAM blocks. While MAK (e-1, 2) had scale-like structures, other glass-ceramic CAD/CAM block had bale-shaped crystal grains. The grain size was measured for 20 arbitrary crystal grains. The length for IPS (b) and DUO (c) were 1.7 µm and 0.6 µm, respectively, whereas LIS (a) and VITS (d) were 0.3 µm or less. Although the grain shape of the MAK was non-uniform and the size could not be measured, large crystals of sizes greater than 5 µm were confirmed.

**Figure 6:**
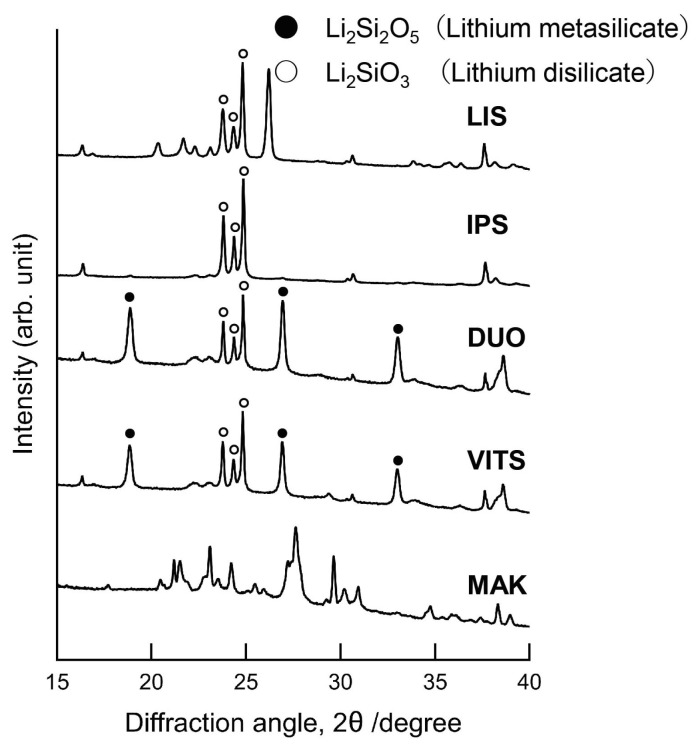
XRD diffraction pattern.

Figure 6 shows the XRD diffraction patterns (Powder XRD patterns) of the glass-ceramic CAD/CAM blocks. The 23.7°, 24.3° and 24.8° peaks found in LIS, IPS, DUO, and VITS were associated with lithium disilicate (ICDD: 40-376). In DUO and VITS, strong peaks of lithium metasilicate (ICDD: 29-829) were confirmed at 18.8°, 26.9°, and 33.0°. In the EPMA analysis, Si, O, C, Al, and K were detected in all materials. Zirconium was detected only in DUO and VITS.

### 3.4. Wear Volume of Abrader Specimen (Glass-Ceramic CAD/CAM Blocks)

Each abrader specimen’s surface roughness (Sa) before the test was approximately 1.50 µm. There was no significant difference between the surface roughness of each glass-ceramic CAD/CAM block (*p* > 0.05).

**Figure 7:**
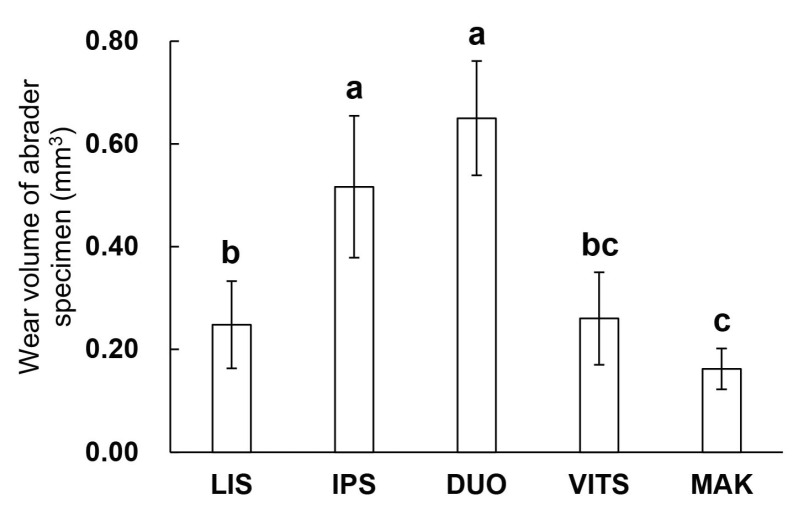
Wear volume of abrader specimen (glass-ceramic CAD/CAM blocks). The same lowercase letters in the figure indicate no significant difference (*p* > 0.05, ANOVA and Tukey’s multiple comparison test).

Figure 7 shows the wear volume of the abrader specimens. The wear volumes ranged from 0.16 mm^3^ to 0.65 mm^3^. As a result of the one-way ANOVA, a difference in the wear volume of the abrader specimens was found depending on the type of glass-ceramics (*p* < 0.05). IPS and DUO had significantly larger abrader wear volumes than LIS, VIS, and MAK (*p* < 0.05). In addition, LIS had a significantly greater abrader wear volume than MAK (*p* < 0.05).

### 3.5. Wear Volume of Substrate Specimen (Antagonist Bovine Enamel)

**Figure 8:**
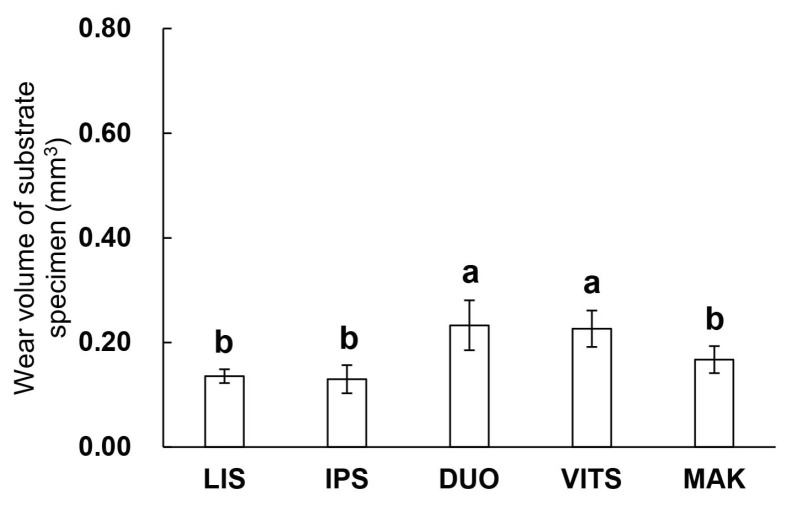
Wear volume of substrate specimen (bovine enamel). The same lowercase letters in the figure indicate no significant difference (*p* > 0.05, ANOVA and Tukey’s multiple comparison test).

Figure 8 shows the amount of wear volume of substrate specimens. The wear volume of the substrates ranged from 0.13 mm^3^ to 0.23 mm^3^. A one-way ANOVA showed that the wear volume of the substrate differs depending on the type of glass-ceramics used for the abrasive (*p* < 0.05). DUO and VITS showed significantly greater wear volume in the substrate compared to the other groups (*p* < 0.05).

### 3.6. SEM Observation after Wear Test

**Figure 9:**
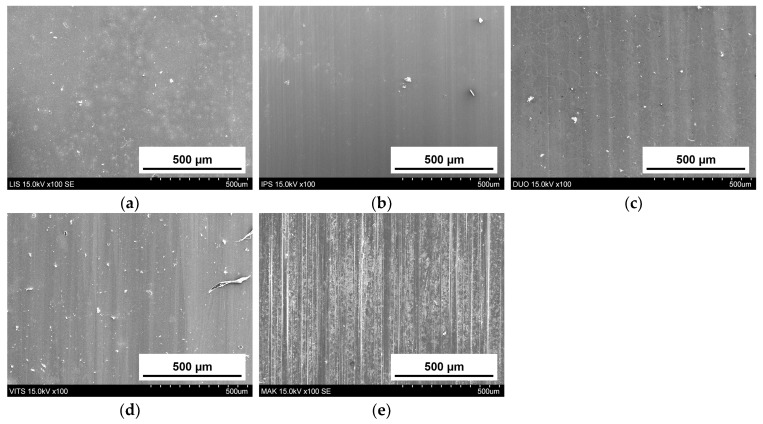
SEM images of abrader specimens after the test (Glass-ceramic CAD/CAM blocks): (**a**) LIS, (**b**) IPS, (**c**) DUO, (**d**) VITS, (**e**) MAK (100× magnification).

**Figure 10:**
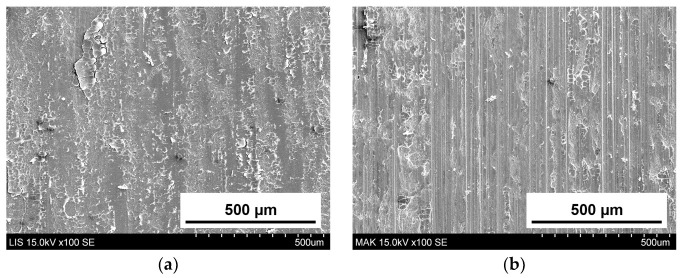
SEM images of substrate specimens after the test (Bovine enamel): (**a**) LIS, (**b**) MAK (100× magnification).

Figure 9 and Figure 10 show the typical SEM images of abrader specimens ((a): LIS, (b): IPS, (c): DUO, (d): VITS, (e): MAK) and substrate specimens ((a): LIS, (b): MAK) after the two-body wear test, respectively. The worn surface on the abrader specimen of LIS, IPS, DUO, and VITS was smooth, whereas scratches that were consistent with the strokes of the wear test were observed on MAK surfaces. In addition, scratches consistent with the strokes of the wear test were confirmed on the substrate specimen for MAK, but were unclear for LIS.

## 4. Discussion

The wear behavior of crown restoration materials is essential when considering the material’s durability and wear on the opposing teeth. Wear tests include the pin-on-disk method and the reciprocating sliding test, which evaluate the wear of crown restorations caused by occluding and grinding [30,31,32,33]. This study conducted a two-body wear test using a reciprocating sliding test to investigate the wear behavior of glass-ceramic CAD/CAM blocks against bovine teeth. Studies have reported that the wear behavior of crown restoration materials and dentin was influenced by the material’s surface roughness (Sa) and mechanical and structural properties [34,35]. In this study, the surface roughness of the abrader specimen was measured before the two-body wear test, and the results confirmed no difference in roughness between the materials. Past studies of wear on enamel have used human or bovine teeth, and bovine teeth were used in this study [16,18,28,29,33,34,35,36]. The previous study suggested that the chemical composition of human teeth and bovine teeth is similar, and bovine enamel is a suitable alternative to human enamel for in vitro testing of dental biomaterials from mechanical and chemical perspectives [37].

The results of a one-way ANOVA after the two-body wear test showed that the type of glass-ceramic CAD/CAM block affects the hardness, three-point bending strength, and wear volume of the abrader and substrate specimens. Therefore, the null hypothesis that the mechanical properties (bending strength, hardness) of the glass-ceramic CAD/CAM blocks do not affect the wear volume of bovine enamel, and the material itself was rejected.

Past studies have reported a correlation between the hardness of the crown restoration material and the wear of the material itself, and the amount of wear can be predicted by the hardness of the material [17,18,36]. In this study, DUO with high hardness showed a large amount of wear volume of the material itself (abrader specimen), and MAK with low hardness showed a small amount of wear volume (Figure 3 and Figure 7). Although the hardness of DUO and LIS were similar, the wear volume of the LIS was small; therefore, no strong correlation was found between the crown restoration material and hardness. These results were consistent with the claim made by Freddo et al. on the relationship between ceramic hardness and wear behavior [38]. However, in their study, the wear behavior was evaluated by wear coefficient and SEM images, which are difficult to directly compare with the amount of wear loss in this study.

Wang et al. reported that differences in the elastic modulus and strength between the materials and enamel caused stress on the surface of the enamel, resulting in enamel wear [22]. The three-point bending strength of the enamel was 100 to 200 MPa, which was not much different from MAK and LIS; however, the two-body wear test showed that all the glass-ceramics wore down bovine enamel (Figure 8) [39]. In addition, LIS had a smaller three-point bending strength than VITS, and the wear volume of the paired enamel was also small. Furthermore, although the bending strengths of IPS and VITS were similar, the wear volume of the enamel was significantly lower for IPS (Figure 4 and Figure 8).

The phase, type, distribution, and shape of crystal grains in dental ceramics affect the wear behavior of materials [17,18,28]. Lithium disilicate at crystal phase was detected in LIS, IPS, DUO, and VITS by X-ray diffraction, and lithium metasilicate was also detected in DUO and VITS (Figure 6). Lithium metasilicate is used as a precursor of lithium disilicate, and this component is crystallized by firing to form lithium disilicate [40]. In VITS (zirconia-reinforced lithium silicate glass-ceramics), the crystal grains of lithium metasilicate grow during the crystallization process [41]. In this study, lithium metasilicate peaks were found in the XRD diffraction patterns of DUO and VITS, and zirconium was detected by EPMA analysis. Since DUO and VITS are partially strengthened as zirconia-reinforced lithium silicate glass-ceramics, these materials significantly caused wear on the antagonist enamel. Zirconia-reinforced lithium silicate glass ceramics are considered to have better mechanical properties than lithium disilicate glass-ceramics [13,14]. However, no clear difference in mechanical properties was observed in this study. It is necessary to evaluate materials focusing on the crystal grains themselves using nanoindentation in the future.

The crystal grains of the glass-ceramic CAD/CAM blocks used in this study were bale-shaped for LIS, IPS, DUO, and VITS and scale-shaped for MAK (Figure 5). DUO and IPS with bale-shaped crystals and relatively large grain sizes showed a large amount of wear in the abrader specimen. Crystal grains detach and fall off during the wear process of glass-ceramic CAD/CAM blocks [18]. In glass-ceramics with bale-shaped crystals, the larger the crystal grain size, the larger the load applied to the crystal grains during wear, causing them to fall off, affecting the abrader specimens’ wear. On the other hand, although large crystal grains were observed in MAK, the material’s wear volume was small. Therefore, it is presumed that the bale-shaped and scale-like crystal grains have different mechanisms of crystal grain shedding due to wear.

After the wear test, scratches consistent with the direction of the test were observed in both the abrader and substrate specimens for MAK (Figure 9 and Figure 10). Although the wear volumes of the substrate were similar for MAK and LIS with scale-like and bale-like crystals, respectively, the surface texture of the worn surfaces was different. Tanaka et al. reported that filler shapes affect the wear of the antagonist enamel in composite materials such as resin composites [36]. Like fillers in the matrix of resin composites, glass-ceramics also have crystals in the amorphous region and can be classified into multiple sectors. The amorphous glass area with low strength was worn during the wear test, and the crystalline area with high strength was exposed, which led to the wear progression. The SEM images (Figure 10) of the surface after the wear test suggested that materials with sharp grain shapes like MAK may increase the amount of wear on the antagonist enamel. However, the wear volume of the substrate for MAK was smaller than that of other glass-ceramics after 30,000 wear strokes, assuming two months of occlusion (Figure 8) [18]. The relationship between the grain shape of glass-ceramics and wear may be better clarified by increasing the stroke number and load during wear tests assuming long-term use of the restoration.

The results of this study indicated that the wear behavior of the glass-ceramic CAD/CAM blocks and antagonist enamel were affected by multiple factors, including crystal composition, shape, and size. On the contrary, a strong correlation between the mechanical properties (hardness, three-point bending strength) and wear volume could not be confirmed. There was no big difference in the mechanical properties of the glass-ceramic CAD/CAM blocks, and the traceability is compensated for when the blocks are shipped from the factory. In the future, in order to determine the effects of the crown restoration material’s mechanical properties on wear behavior, zirconia with significantly different strength and conventional ceramics (which are layered and fired) to demonstrate the usefulness of the glass-ceramic CAD/CAM blocks.

In this study, LIS and MAK were considered wear-friendly materials because the wear volume of both the abrader and substrate specimens was small. On the other hand, feldspar-based glass-ceramics, such as MAK, had low strength, and reports have suggested that the material often fractures when used as crowns and that the range of use was limited [41]. Factors such as mechanical strength, wear, and aesthetics must be considered when selecting materials for CAD/CAM blocks.

## 5. Conclusions

Within the limitations of this study, the conclusion are as follows:

1. Hardness of glass ceramics for CAD/CAM blocks did not affect the wear behavior with bovine enamel;
2. Three-point bending strength of glass ceramics for CAD/CAM blocks did not affect the wear behavior with bovine enamel;
3. Zirconia-reinforced lithium silicate glass-ceramics significantly abraded the antagonist bovine enamel compared to lithium disilicate glass-ceramics and the feldspar-based glass-ceramics;
4. Glass-ceramics with scale-shape crystals grains produced distinct scratches after wear test in the material itself and the antagonist bovine enamel.

Thus, dentists should consider that wear behavior varies with crystal structure, size, and shape in glass-ceramics for CAD/CAM blocks.

## Figures and Tables

**Table 1:** Types of glass-ceramic CAD/CAM blocks used.

Material		Manufacturer	Lot Number	Code
Lithium disilicate glass-ceramic				
	Initial LiSi block	GC, Tokyo, Japan	1902121	LIS
	IPS e.max CAD	Ivoclar-Vivadent, Tokyo, Japan	Y07913	IPS
Zirconia-reinforced lithium silicate glass-ceramic				
	Celtra DUO	Dentsply Sirona K.K., Tokyo, Japan	16004292	DUO
	VITA Suprinity	VITA, Bad Säckingen, Germany	47558	VITS
Feldspar-based glass-ceramic				
	Vitablocs MarkII	VITA, Bad Säckingen, Germany	77970	MAK

## Data Availability

All data are included in the manuscript.
